# Determining factors of presentation and diagnosis delays in patients with colorectal cancer and the impact on stage: a cross sectional study in Yogyakarta, Indonesia

**DOI:** 10.3332/ecancer.2024.1761

**Published:** 2024-09-11

**Authors:** Norma Dewi Suryani, Juan Adrian Wiranata, Herindita Puspitaningtyas, Susanna Hilda Hutajulu, Yayi Suryo Prabandari, Adeodatus Yuda Handaya, Mardiah Suci Hardianti, Kartika Widayati Taroeno-Hariadi, Johan Kurnianda, Ibnu Purwanto

**Affiliations:** 1 Clinical Epidemiology Study Program, Master of Clinical Medicine Postgraduate Program, Faculty of Medicine, Public Health and Nursing, Universitas Gadjah Mada, Yogyakarta 55281, Indonesia; 2 Academic Hospital, Universitas Gadjah Mada, Yogyakarta 55281, Indonesia; 3 Doctorate Program of Health and Medical Science, Faculty of Medicine, Public Health and Nursing, Universitas Gadjah Mada, Yogyakarta 55281, Indonesia; 4 Division of Hematology and Medical Oncology, Department of Internal Medicine, Faculty of Medicine, Public Health and Nursing, Universitas Gadjah Mada, Dr. Sardjito General Hospital, Yogyakarta 55281, Indonesia; 5 Department of Health Behaviour, Environment, and Social Medicine, Faculty of Medicine, Public Health and Nursing, Universitas Gadjah Mada, Yogyakarta 55281, Indonesia; 6 Center of Health Behaviour and Promotion, Faculty of Medicine, Public Health and Nursing, Universitas Gadjah Mada, Yogyakarta 55281, Indonesia; 7 Department of Surgery, Faculty of Medicine, Public Health and Nursing, Universitas Gadjah Mada, Yogyakarta 55281, Indonesia

**Keywords:** colorectal neoplasms, delayed diagnosis, neoplasm metastasis

## Abstract

**Background:**

Early colorectal cancer (CRC) symptom recognition and prompt diagnosis are crucial for the identification of cases in the earliest stage and for improving survival. This study investigates the incidence of presentation and diagnosis delays, their contributing determinants and their impact on the cancer stage at diagnosis.

**Methods:**

This cross-sectional study recruited 227 CRC patients between November 2022 and October 2023. We developed a semi-structured questionnaire to collect information on the factors related to delays in the presentation and diagnosis. Presentation delay was defined as the time between the initial symptoms and the first consultation exceeding 1 month, while diagnosis delay was defined as the time between presentation and the pathological diagnosis confirmation exceeding 4 months. We examined the impact of these delays on the status of the metastatic disease and identified the determinants of the presentation and diagnosis delays.

**Results:**

The median values for presentation and diagnosis delay are 1 and 4 months, respectively. Patients aged ≥60 years were less likely to experience diagnosis delays odds ratio (OR = 0.52, 95% confidence interval (CI) 0.28–0.95, *p* = 0.035), as opposed to those who were younger. The absence of red flag symptoms at presentation (OR = 2.73, 95% CI 1.47–5.10, *p* = 0.002), the utilisation of complementary and alternative medicine (OR = 2.01, 95% CI 1.12–3.61, *p* = 0.019) and ≥3 distinct healthcare facility visits before diagnosis (OR = 3.51, 95% CI 1.95–6.29, *p* < 0.001) were associated with an increased risk of diagnosis delays. Diagnosis delays were also correlated with a higher risk of metastatic disease at diagnosis (OR = 2.04, 95% CI 1.17–3.53, *p* = 0.011).

**Conclusion:**

Our CRC patients experience considerable delays in their presentation and diagnosis. Diagnosis delays were observed to increase the likelihood of presenting with metastatic disease. Given the determinants and the patients’ perspectives revealed in this study, future research to explore evidence-based approaches to reducing these delays is warranted.

## Background

Colorectal cancer (CRC) ranks as the third most common cancer globally, and the second leading cause of cancer-related deaths [1]. In 2020, CRC ranked fourth in terms of incidence, accounting for approximately 8.6% of all new cancer cases in Indonesia [2]. According to data from the hospital-based cancer registry of the top referral hospital in Yogyakarta, Indonesia, CRC is the second most frequently encountered cancer among patients, after breast cancer. The majority of CRC cases are diagnosed at stages III–IV [3].

Recent studies have explored the impact of delay in the presentation and diagnosis of CRC, which profoundly impacts patient outcomes. Patients with CRC have unique characteristics upon presentation, as this population might present with nonspecific symptoms. Given that most cases are suspected based on initial symptoms [4], patients with nonspecific symptoms can have prolonged presentation to healthcare facilities and experience misdiagnosis within the healthcare facilities [5–7]. The limitation in our country’s screening capabilities might impede prompt diagnosis and treatment, which also hinders treatment commencement, which is crucial for improving prognosis [8, 9]. Delays in the presentation and diagnosis of CRC patients have been shown to affect the stage at presentation [10], which might also limit treatment options, lead to poorer survival [11] and possibly contribute to the economic burden on the healthcare system [12].

Numerous factors may contribute to delays in seeking healthcare and the prompt diagnosis of CRC. These include sociodemographic factors, access to health services, knowledge of CRC, the use of herbal remedies and visits to alternative medicine practitioners, the type of initial symptoms, the family’s history of cancer and the tumour’s location [13–16]. Identifying these determinants may help make improvements in the future by identifying certain patient characteristics that could cause delays.

Indonesian patients may have different characteristics that could lead to unique patterns and causes of presentation and diagnostic delays compared to those in other countries. Besides factors observed in the literature, various aspects such as cultural beliefs, perceptions regarding cancer and health-care-seeking behaviours may influence the delays. However, only a few studies have explored delays in either the presentation or diagnosis of CRC cases [17, 18], with the causal factors not being well known. Therefore, this study aims to determine the occurrence of delayed presentation and diagnosis in local CRC patients, identify the contributing factors and explore the correlation between these delays and the disease stage at diagnosis.

## Methods

### Study design and participants

This study is a cross-sectional study that recruited patients from the Hematology and Medical Oncology Division, ‘Tulip’/Integrated Cancer Clinic, Dr. Sardjito General Hospital, Yogyakarta, Indonesia, between November 2022 and October 2023. The subjects were recruited using consecutive sampling, whereby members of the research team approached patients attending the clinic. These individuals were provided with detailed explanations regarding the study, and written informed consent was obtained from each subject. The subjects included were patients aged 18 or older with histopathologically confirmed primary CRC. Subjects with terminal illness, severe comorbidities or patients unable to give proper history due to psychotic illness or any other reason were excluded from the study. Exclusion was done to mitigate potential limitations in recall ability and interview coherence and to prevent unwanted harm to these particular groups who might have experienced distress regarding their condition.

### Questionnaire development and delivery

A semi-structured questionnaire was developed to gather information on the factors related to the delays in the presentation and diagnosis of CRC. The questionnaire was adapted from a previous study which investigated delays in the presentation and diagnosis of breast cancer patients [5]. Further modifications were made by incorporating elements from various sources, including the Indonesian Family Life Survey Wave 5 for information on living arrangements, socioeconomic status and accessibility to the initial medical facility visit [19]. Additionally, content from the Bowel Cancer Awareness Measure toolkit version 2.1 was integrated to assess the patients’ knowledge regarding CRC symptoms and risk factors, aligning the questionnaire with the specific context of CRC in this study [20].

In the questionnaire’s development phase, two practicing general practitioners conducted a forward-and-backward translation into Bahasa Indonesia. In addition to the questionnaire, a script outlining a stepwise interview procedure was created, to guide the interviewers during the data collection phase. Subsequently, the developed questionnaire underwent a face validity evaluation, and a pilot test was carried out using ten individuals with no medical background. A medical oncologist also reviewed the final version of the questionnaire and the interviewers’ guidance script. Face-to-face interviews were then conducted by trained members of the research team.

### Study variables

Data regarding the patients’ characteristics were obtained from the medical records and through interviews at baseline. The sociodemographic data included age, sex, monthly income, formal education attainment, marital status, living arrangements, insurance status at first presentation, insurance status following presentation, number of health facilities visited before diagnosis, distance to the first health facility visited and the consumption or use of complementary and alternative medicine (CAM) before presentation and before diagnosis (which also included usage before presentation). The subjects were also asked an open-ended question about the delayed consult to a healthcare facility.

The subject’s age was defined as the age at CRC diagnosis, and further dichotomized into groups of patients who were less than or equal to 60 years old, and elderly group whose age was greater than 60. Monthly income was defined as the individual or household earnings on a monthly basis, dichotomized based on the median value. Formal education attainment refers to the highest level of education that the individual completed within the formal education system, categorised into subjects with the educational attainment of a higher degree (bachelor’s degree or higher), junior to senior high school and primary school or no formal education attained. Marital status refers to an individual’s legal relationship status in terms of marriage, dichotomized into married, single, widowed or divorced. Living arrangements describe the composition of people residing together in a particular household, dichotomized into with spouse or without a spouse (with others such as parents, children, relatives or alone).

Insurance status at first presentation refers to the subject’s insurance coverage or lack thereof, at the initial point of presenting themselves for healthcare services, dichotomized into using national or private health insurance or out-of-pocket payment. The insurance status following the presentation refers to insurance coverage or changes in the coverage that occurred after the initial presentation for healthcare services. This includes any alterations, additions, or cancellations in the individual’s insurance status that may have occurred while seeking treatment, dichotomized into non-incentivized national or private insurance or government-incentivized national health insurance.

The distance to the first health facility visited refers to the geographical distance (in kilometres) between the individual’s residence or location at the time of seeking medical attention, and the healthcare facility they visited at their first presentation, and is dichotomized based on its median values. The number of health facilities visited before diagnosis refers to the number of distinct medical facilities or healthcare providers that an individual consulted for the evaluation, assessment or treatment before receiving a pathologically confirmed diagnosis of CRC. This variable was dichotomized into less than or equal to three health facilities or greater than three facilities. The rationale of this dichotomization is that in Indonesia, the healthcare system guides patients through a network of facilities, ranging from primary care centres to specialised hospitals. Typically, patients undergo pathological diagnostic procedures at tertiary-level referral hospitals after initial consultations at primary care centres. This pattern represents the prevalent and anticipated trajectory of healthcare-seeking behaviour within the framework of Indonesia’s national healthcare insurance coverage. The clinical variables included were the family history of any malignancy, the family history of CRC, number of red flag symptom(s) at presentation and the stage at diagnosis. Family history of any malignancy and CRC did not exclusively include first-degree relatives, dichotomized into yes and none. Early symptoms of CRC include the presence of bloody stools or rectal bleeding, a change in bowel habits (diarrhoea and constipation), abdominal mass, rectal mass, bloating or tenesmus, weight loss, abdominal pain, rectal pain and fatigue. Red flag symptoms were defined according to previous studies, and included abdominal pain, rectal bleeding and diarrhea [21]. The stage at diagnosis was defined based on the 8th edition of the AJCC cancer staging system [22] and dichotomized into stages I–IV.

### Definition of delays in presentation and diagnosis

The time frame between the onset of symptoms related to CRC and the subsequent diagnosis was divided into two distinct periods. The presentation duration is the interval between the initial onset of symptoms related to CRC and the patient’s first consultation with a medical professional, such as a doctor, nurse or midwife. On the other hand, the duration of the diagnosis is characterised as the period between the first medical visit and the date of receiving the first pathology report confirming a CRC diagnosis. The date of diagnosis was retrieved from the date of the pathological report of CRC.

Regarding the date of the symptoms’ onset, when the participants could not recall specific dates for their symptoms’ onset or the first medical visit, they were prompted to provide a time period in months. Following this, efforts were made to assist the participants in refining the time frame by linking it to significant dates or events, such as family birthdays, religious holidays or other notable occasions. This aimed to enhance their recall precision and narrow the range closer to the exact date.

If the respondents provided a single month, additional inquiries were made to ascertain the exact day, or its proximity to other significant events within that month, thereby further refining the date. In cases where surgery or colonoscopy procedure dates were documented in clinical notes, they were used as benchmarks, as they were commonly considered important events by most patients. Then, participants were explicitly asked about the dates of the first symptoms and the initial medical visit, with the surgery or colonoscopy date as a benchmark. When the respondents still could not point to a specific date for their symptoms’ onset, or the first provider visit, they were requested to provide a month or a range of months along with the year. If a month was provided, the estimated date was the 15th of that month. In cases of months range, the midpoint between the 15th of those months was used as the estimated date. If only the year was provided, the estimated date would be set as June 30 of that particular year [5, 23].

From the presentation and diagnosis duration data, delay variables were defined by dichotomizing these variables with their median values. Notably, the diversity in CRC screening and diagnostic pathways and the unique healthcare systems constructed across different populations may introduce heterogeneity. Adopting a cutoff established from another study population may not accurately reflect the typical delay experienced by patients in our specific setting. Therefore, utilising the median for delay determination from our dataset offers a more precise assessment tailored to our research question within our local setting.

### Statistical analysis

Data on the subjects’ characteristics were presented as either the median, mean and standard deviation (SD) or as frequencies. Bivariable logistic regression analysis was then used to assess the sociodemographic and clinical variables associated with the presentation and diagnosis delays accordingly. Variables with a *p*-value of <0.250 were further analysed using multivariable logistic regression analysis. A multivariable logistic regression was also employed to assess the association of the presentation and diagnosis delays with the metastatic disease status at diagnosis, with a *p*-value of <0.05 deemed statistically significant. A content analysis of the interviews’ transcripts [24] was conducted to evaluate the reasons for the patients’ delayed presentation. The transcripts were systematically coded through open coding, wherein responses were comprehensively categorised to encompass all the pertinent data and derive meaningful insights. For our supplementary analysis, we investigated the differences in the durations of the presentation and diagnosis (measured in days), based on various patient characteristics, using the Wilcoxon rank-sum test and the Kruskal-Wallis test, with a *p*-value of <0.05 being statistically significant. All the statistical analyses were conducted using STATA software version 17 (Stata Corp., College Station, TX).

## Results

The subjects’ baseline characteristics are presented in Table 1. Out of the 234 patients initially registered for this study, 17 were excluded due to missing or incomplete data, resulting in 227 patients being included in the analysis. The mean age of the study’s participants was 56.0 years. The majority of the participants were female (55.9%), reported a monthly income of ≤2,000,000 IDR (66.5%), attained formal education up to junior or senior high school (41.9%), were married (82.8%) and resided with their spouses (81.5%).

Before their initial presentation, only a minority of patients (11.0%) disclosed that they were consuming or utilising CAM for symptoms related to CRC. Most patients (52.9%) lived no more than 3.8 km from the first healthcare facility they visited. Upon their first presentation, slightly more patients (52.0%) utilised national or private health insurance compared to those who paid out-of-pocket, and the majority (65.6%) did not exhibit red flag symptoms.

Prior to receiving a confirmed pathological diagnosis of CRC, the percentage of patients acknowledging the use of CAM increased to 44.1%, encompassing the periods both before the presentation and before the diagnosis. The majority of patients (53.3%) sought care from more than three healthcare facilities before obtaining a confirmed diagnosis. Throughout the diagnostic and treatment processes, all the patients (100%) utilised insurance coverage, with the majority relying on either non-incentivized national or private health insurance (52.0%). The majority of patients (56.4%) were diagnosed at stages I–III.

The median duration from the symptoms’ onset to the patient’s first presentation was 31 days, leading to the selection of a rounded cutoff of 1 month for defining the presentation delay, whereby 114 patients (50.2%) delayed their presentation by ≥1 month. The median duration for diagnosis was 118 days, prompting the selection of a rounded cutoff of 4 months for defining the diagnosis delay, with 112 patients (49.3%) experiencing a delay in diagnosis of ≥4 months (Table 1).

### Factors associated with presentation and diagnosis delay

Table 2 presents the sociodemographic and clinical factors associated with the presentation delay among the subjects. In the multivariable analysis, no statistically significant factors were identified. Male subjects are shown to have a higher risk of presentation delay compared to their female counterparts, although the association demonstrates marginal significance (odds ratio (OR) = 1.68, 95% confidence interval (CI) 0.99–2.88, *p* = 0.056). However, in terms of the presentation duration, it is evident that males significantly consult at a later time after initial symptoms than their female counterparts (median of 46 versus 23 days, respectively, *p* = 0.038) (Supplementary Table 1).

Table 3 displays the sociodemographic and clinical factors associated with the diagnosis delay. In the multivariable analysis, elderly subjects exhibited a statistically significant lower risk of experiencing a diagnosis delay, compared to their younger counterparts (OR = 0.52, 95% CI 0.28–0.95, *p* = 0.035). Additionally, presenting with no red flag symptoms, consuming or using CAM before diagnosis and visiting more than three distinct healthcare facilities before diagnosis were significantly associated with a higher risk of diagnosis delay (OR = 2.73, 95% CI 1.47–5.10, *p* = 0.002; OR = 2.01, 95% CI 1.12–3.61, *p* = 0.019; OR = 3.51, 95% CI 1.95–6.29, *p* < 0.001, respectively). Regarding the duration of the diagnosis, it was evident that elderly patients experienced shorter durations, compared to their younger counterparts (median of 90 versus 151.5 days, respectively; *p* = 0.005). Similarly, patients presenting with no red flag symptoms (median of 148 versus 89 days; *p* = 0.005), those utilising or consuming CAM before diagnosis (median of 181 versus 90 days; *p* < 0.001), and those visiting more than three distinct health facilities before diagnosis (median of 182 versus 71 days; *p* < 0.001) tended to have longer diagnosis durations. Patients with a family history of CRC exhibited longer diagnosis durations (median of 272.5 versus 110 days; *p* = 0.007) (Supplementary Table 2).

### The association between presentation and diagnosis delay with metastatic disease at diagnosis

Table 4 presents the association between delays and the metastatic disease status at diagnosis. Following adjustments for age, gender, monthly income and formal education level, no significant association was observed between the presentation delay and the metastatic disease status at diagnosis. Further, patients experiencing a diagnosis delay of ≥4 months exhibited a significantly higher risk of being diagnosed with metastatic disease (OR = 2.04, 95% CI 1.17–3.53, *p* = 0.011) compared to their counterparts, after adjustments for age, gender, monthly income, formal education level and presentation delays. Patients with metastatic disease status at diagnosis also had a significantly longer diagnosis duration (median of 154 versus 93.5 days; *p* = 0.005) (Supplementary Table 2).

### Reasons for delaying presentation

Table 5 illustrates the findings of a content analysis conducted on interview transcripts concerning the reasons why 114 patients waited for 1 month or more before their first visit to a healthcare facility for CRC symptoms. Among them, 44 patients perceived no delay in presenting themselves to a healthcare facility. For the 70 patients who perceived some delay, the primary reasons identified for their delayed presentations were the belief that their condition was benign and could potentially resolve itself on its own (48.6%), the fear of consulting a physician or visiting a healthcare facility (17.1%) and the absence of noticeable pain associated with the symptoms (14.3%). The content analysis for the reasons behind the delayed presentation for the whole cohort of 227 patients is presented in Supplementary Table 3.

## Discussion

This study is the first in Indonesia to depict the delays in the presentation and diagnosis of CRC patients, and the resulting clinical impact. Given Indonesia’s distinctive healthcare and cultural landscape, which significantly influences healthcare-seeking behaviours, these findings provide valuable insights for patients, clinicians and decision makers into the challenges patients and healthcare systems face in resource-constrained settings.

### Summary of key findings

The median values for presentation and diagnosis delay are 1 and 4 months, respectively. Elderly patients aged 60 years or more are less likely to experience diagnosis delays. Other factors such as the absence of red flag symptoms at presentation, the utilisation of CAM and three or more distinct healthcare facility visits before diagnosis are associated with an increased risk of diagnosis delays. Notably, diagnosis delays are associated with the presence of metastatic disease. The primary reasons for presentation delays include a belief that the condition is benign and may resolve itself spontaneously, the fear of seeking medical advice or visiting a healthcare facility and the absence of noticeable pain associated with the symptoms.

### Comparison with previous findings

In terms of the presentation duration, our study’s population exhibited a median presentation duration of 31 days, which was longer than a study in Spain found (19 days) [25], but comparable to The Netherlands (30 days) [16] and slightly shorter than Australia (41 days) [26]. A median diagnostic duration of 118 days was longer compared to a study in Spain (66 days) [25], Canada (89 days) [27] and Australia (49 days) [26]. These differences in duration may arise from varying socioeconomic profiles and healthcare policies. Compared to previous pooled meta-analysis results, the presentation duration observed in this study was shorter than the median duration of 53 days reported in lower income countries and comparable to the median of 26 days found in higher income countries. However, the diagnosis duration in our study was significantly longer compared to previous findings, with a median of 59 days in higher income countries and 46 days in lower income countries [28], highlighting the importance of improvement in diagnostic capability and referral pathway within our local population. The information regarding the duration of the delay could serve as a benchmark for informing policymakers about the current status of delays in CRC cases in Indonesia, and potentially lead to the identification of successful strategies in other centres that could be adapted to local contexts to generate improvements.

We also revealed notable differences in the presentation and diagnosis durations between CRC in our study and from breast cancer cohorts from a previous study in Yogyakarta [5]. In terms of the presentation duration, CRC exhibited a shorter median duration of 1 month, compared to breast cancer’s median duration of 2 months. Individuals with CRC might experience symptoms such as blood in their stools, rectal bleeding, changes in their bowel habits and other symptoms that might be perceived as more alarming and bothering, prompting quicker action compared to the relatively subtle change in breast tissue in breast cancer patients. Such disparities in presentation durations underscore the varying symptomatology and awareness levels associated with the types of malignancies within the patient population. However, the diagnostic journey for CRC patients unveils contrasting dynamics. Despite presenting sooner, they experienced a longer median diagnosis duration of 4 months, compared to those with breast cancer who had a 1-month median duration. This protracted diagnostic timeline for CRC patients may stem from multifactorial challenges within the healthcare system, including the complexity of the diagnostic procedures and accessibility issues. Interestingly, CRC and breast cancer patients share problems of delays in the referral pathways [5]. Colonoscopies, often used for CRC diagnosis, are not readily available in Indonesia. This can lead to longer wait times for scheduling and performing the procedure. Such findings underscore the critical need for streamlining the diagnostic processes and addressing systemic barriers to expedite the timely diagnosis of CRC.

We found no significant determinants of presentation delays exceeding 1 month. However, we observed that males exhibited longer durations compared to their female counterparts (Supplementary Table 1). This difference in health-seeking behaviour affecting the presentation delay in males has also been noted in previous studies [29–31]. The delay in healthcare presentation among men can be attributed to various sociocultural factors. Traditionally, men are perceived as less knowledgeable about health issues and often rely on partners or female family members as a role of caretakers [32, 33], and typically justify consulting a doctor only when symptoms interfere with their work [34]. Men also face social pressure to conform to norms of independence and control, which discourages seeking medical help, seen as a sign of weakness [35, 36]. Additionally, men have fewer confidantes and mainly discuss health issues with their partners, making it more difficult for them to cope with serious illnesses such as cancer [32, 37]. A previous study in Indonesia also confirmed that men have a lower rate of primary healthcare visits compared to women [38].

In our study, we identified several determinants of diagnosis delays. A lower risk of diagnosis delays in elderly patients was consistent with those found in other studies. This could be related to clinicians potentially having a lower threshold for conducting further investigations in this population [39, 40]. The absence of red flag symptoms was associated with diagnosis delays, potentially prolonging the investigative process. Patients with nonspecific symptoms may be misdiagnosed and excluded from case prioritization [5–7]. The utilisation of CAM as a diagnosis delay determinant highlighted the observations made in previous studies [41, 42]. In Indonesia, where many patients are afraid or reluctant to consult formal health facilities, there is a significant portion of the population favours CAM in the hope of curing cancer, thus prolonging the diagnostic process [17]. Furthermore, frequent visits to different health facilities after presentation were consistent with previous findings, in terms of its relationship with a higher risk of diagnosis delays [5, 43–45]. Regarding this issue, we only explored patient-level determinants and did not particularly investigate the factors causing health service delays. The major determinants of system-oriented delays include the failure of general physicians to recognize and work on clinical manifestations, maltreatment or prolonged treatment of the clinical signs as a benign disease, false negatives or misinterpretation of the early screening results such as faecal occult bleeding or colonoscopy and false-negative results of cytology [46–48]. These, nevertheless, warrant future investigation that can lead to improvement initiatives. In the literature context, the delay in diagnosis can be anticipated through the delivery of efficient training programs for medical personnel [49]. Our findings also specify a necessity for simpler and more efficient referral systems for patients suspected of having cancer to access health facilities providing cancer management. Interestingly, patients with a family history of CRC had a higher diagnosis duration (Supplementary Table 2). This could be attributed to the fear of treatment and its possible side effects, possibly influenced by experiences observed in other family members diagnosed with CRC. This finding is consistent with previous research on diagnosis delays in our local breast cancer patients, suggesting unique health-seeking behaviour patterns in our population [5].

In this study, a significant association between delayed presentation and the presence of metastatic disease was not observed, which supports a previous study in Jordan [13]. On the contrary, we found a notable association between the delay in diagnosis and metastatic disease. Studies showed that shorter diagnostic intervals have been linked to earlier stage diagnosis in rectal cancer cases [50]. The likelihood of advanced CRC also increases with longer primary care intervals (up to 90 days) [51] and a prolonged diagnostic interval is associated with higher mortality rates [52]. However, the results regarding whether longer diagnostic intervals have a linear association with poorer stage and survival outcomes remain inconclusive. Previous research has highlighted a ‘wait time paradox’ in diagnostic delays, where patients with very short waiting times are often diagnosed with more advanced diseases and experience poorer outcomes [51]. Several confounding factors have been implicated in this paradox. These include tumour aggressiveness [53], admission through emergency departments [54] and ‘confounding by indication’. These all lead to follow-up testing prioritization for more severe screening findings or more seriously ill patients or those who present with alarming symptoms [55]. To confirm this waiting time paradox, further research is needed to investigate the implications of diagnostic delays in the presenting stage and survival of CRC patients, including patients with very short diagnostic intervals in our local population.

In patients who delayed their presentation for more than 1 month, the primary reasons identified were the belief that their condition was benign and could resolve itself independently, the fear of consulting a physician or visiting a healthcare facility and the absence of noticeable pain associated with their symptoms. These reasons are similar to those found in our previous observation on local breast cancer cases [5] and other Indonesian studies of CRC patients [18]. In our country, health-related behaviour is influenced by its multicultural and ethnically diverse context and the variety of health providers available. People in Indonesia consider themselves healthy if they can perform their daily activities without disruption, seeking help only when their symptoms hinder them. Some individuals also perceive that medicine is inadequate for curing illnesses. Fear of the medication’s side effects, the possibility of dependence on the medication, and the fear of medical procedures are common reasons cited for avoiding formal healthcare facilities [56]. These behaviours may also be influenced by knowledge and awareness levels. Indonesia was observed to have lower awareness than other Asia-Pacific countries regarding CRC risk factors, symptoms and screening tests [55, 57, 58]. Additional education programs to address these issues are recommended, to increase the public’s awareness and education regarding CRC.

### Study strengths and limitations

Our study exhibits several strengths in its investigation of presentation and diagnosis delays. The administration of semi-structured questionnaires by trained research personnel during interviews enables the facilitation of the patients’ diverse educational and cultural backgrounds, compared to using a checklist format. Furthermore, the interview sessions were conducted not long after the patients were diagnosed, to help mitigate any recall bias. The integration of various sociodemographic factors enabled a thorough exploration of the determinants of any delay. This study also incorporates patients’ perspectives regarding the reasons for presentation delays, which might give insights into the multifaceted nature of the delay in seeking medical attention.

The present study also has some limitations. Cross-sectional study design was chosen because the baseline patient’s factor and the presentation and diagnosis delay data were collected simultaneously at a time (after CRC diagnosis). Such design aims to find association and may not be inferred as investigating causality. Although causality cannot be directly proven, it aligns with the study objective wherein identifying delay-related factors can inform future research and potentially guide interventions. The study was carried out in a single centre with a consecutive sampling method, which may inevitably introduce selection bias and require a careful generalisation of our findings. Additionally, the exclusion of patients with terminal illnesses and severe comorbidities may introduce a degree of selection bias, potentially influencing the observed outcomes. Furthermore, the inherent challenge of recall bias regarding the date of the onset of any symptoms is acknowledged. However, as outlined in the methodology section, we employed methods to mitigate this bias to the best of our ability. Another limitation is that the majority of patients underwent staging at the study site, a tertiary referral hospital. This results in time intervals between diagnostic confirmation at secondary referral hospitals and the stage’s establishment. During this interval, some cases may have progressed in their stage. A 1-month interval is considered acceptable for staging establishment after diagnosis [59]. In our study, the median interval time between diagnosis confirmation and the stage’s establishment was 52 days, with 46.3% of patients experiencing an interval of more than a month.

## Conclusion

Our CRC patients experience considerable delays in their presentation and diagnosis, in which diagnosis delays increase the likelihood of presenting with metastatic disease. Although we did not find any factors contributing to the delayed presentations, we identified the primary reasons behind the presentation delays, including the low perceived severity, the fear of consultations with health personnel and painless symptoms. Old age is a significant factor associated with the lower risk of diagnosis delays, while the absence of red flag early symptoms, the use of CAM and frequent visits to distinct healthcare facilities before diagnosis are significant determinants for diagnosis delays. These findings offer valuable insights into the local context, for identifying areas for improvement and also serve as benchmarks for the global context. Further investigation is warranted to explore evidence-based approaches to reducing these delays.

## Conflicts of interest

The author(s) declare that they have no conflict of interest.

## Ethical approval and consent to participate

This study has received ethical approval from the joint ethics committee of the Faculty of Medicine, Public Health, and Nursing of Universitas Gadjah Mada/Dr. Sardjito General Hospital (reference number: KE/FK/0953/EC/2022 and KE/FK/1474/EC/2023). Written informed consent was obtained from all the subjects enrolled in the study.

## Author contributions

Conception and design: Norma Dewi Suryani, Juan Adrian Wiranata, Herindita Puspitaningtyas, Susanna Hilda Hutajulu, Yayi Suryo Prabandari

Administrative support: Norma Dewi Suryani, Herindita Puspitaningtyas, Susanna Hilda Hutajulu

Provision of study materials or patients: Norma Dewi Suryani, Herindita Puspitaningtyas, Susanna Hilda Hutajulu, Adeodatus Yuda Handaya, Mardiah Suci Hardianti, Kartika Widayati Taroeno-Hariadi, Johan Kurnianda, Ibnu Purwanto

Collection and assembly of data: Norma Dewi Suryani, Herindita Puspitaningtyas

Data analysis and interpretation: Norma Dewi Suryani, Juan Adrian Wiranata, Herindita Puspitaningtyas, Susanna Hilda Hutajulu, Yayi Suryo Prabandari, Adeodatus Yuda Handaya, Mardiah Suci Hardianti

Manuscript writing: Norma Dewi Suryani, Herindita Puspitaningtyas, Juan Adrian Wiranata, Susanna Hilda Hutajulu, Yayi Suryo Prabandari, Adeodatus Yuda Handaya, Mardiah Suci Hardianti, Kartika Widayati Taroeno-Hariadi, Johan Kurnianda, Ibnu Purwanto

Final approval of manuscript: Norma Dewi Suryani, Herindita Puspitaningtyas, Juan Adrian Wiranata, Susanna Hilda Hutajulu, Yayi Suryo Prabandari, Adeodatus Yuda Handaya, Mardiah Suci Hardianti, Kartika Widayati Taroeno-Hariadi, Johan Kurnianda, Ibnu Purwanto

## Figures and Tables

**Table 1.** Subjects’ baseline characteristics (*n* = 227).

Variables	Frequency (%)/mean ± SD/median (min–max)
Age at diagnosis	56.0 (22.2–89.2)
≤60 years	144 (63.4)
>60 years	83 (36.6)
Sex	
Female	127 (55.9)
Male	100 (44.1)
Monthly income (IDR)	
≤2,000,000	151 (66.5)
>2,000,000	76 (33.5)
Formal education attainment	
Higher degree	60 (26.4)
Junior to senior high school	95 (41.9)
Primary school or none	72 (31.7)
Marital status	
Married	188 (82.8)
Single/widowed/divorced	39 (17.2)
Living arrangement	
With spouse	185 (81.5)
Without spouse (with other or alone)	42 (18.5)
Family history of malignancy	
No	156 (68.7)
Yes	71 (31.3)
Family history of CRC	
No	199 (87.7)
Yes	28 (12.3)
CAM consumption/service before presentation	
No	202 (89.0)
Yes	25 (11.0)
Distance to the first health facility visited (km)	3.8 (0–444)
≤3.8	120 (52.9)
>3.8	107 (47.1)
Insurance status at first presentation	
National/private health insurance	118 (52.0)
Out-of-pocket	109 (48.0)
Number of red flag symptom(s) at presentation	
≥1 symptom(s)	78 (34.4)
No red flag symptom	149 (65.6)
CAM consumption/service before diagnosis	
No	127 (55.9)
Yes	100 (44.1)
Number of health facility visits before diagnosis	4 (1–10)
≤3 health facility(-ies)	106 (46.7)
>3 health facilities	121 (53.3)
Insurance status following presentation	
Non-incentivized/private	125 (55.1)
Government-incentivized	102 (44.9)
Stage at diagnosis	
I–III	128 (56.4)
IV	99 (43.6)
Presentation duration (days)	31 (0–2092)
≤1 month delay	113 (49.8)
>1 months delay	114 (50.2)
Diagnosis duration (days)	118 (3–5088)
≤4 month(s) delay	115 (50.7)
>4 months delay	112 (49.3)

Abbreviations: SD = standard deviation; Min = minimum; Max = maximum; CAM = complementary and alternative medicines

**Table 2.** Univariate and multivariate analyses of factors associated with presentation delay of ≥1 month in patients with colorectal cancer (*n* = 227).

Variables	Presentation Delay >1 Months (%)	Unadjusted OR (95% CI)	p-value	Adjusted OR (95% CI)	p-value
Age at diagnosis					
<60 years	72 (63.2)	Ref			
≥60 years	42 (36.8)	1.02 (0.60–1.76)	0.930		
Sex					
Female	57 (50.0)	Ref		Ref	
Male	57 (50.0)	1.63 (0.96–2.76)	0.071	1.68 (0.99–2.88)	0.056
Monthly income (IDR)					
≤2,000,000	66 (57.9)	Ref		Ref	
>2,000,000	48 (42.1)	0.71 (0.42–1.21)	0.208	0.65 (0.38–1.12)	0.120
Formal education attainment					
Higher degree	27 (23.7)	Ref			
Junior–senior high school	51 (44.7)	1.42 (0.74–2.71)	0.293		
Primary school or none	36 (31.6)	1.22 (0.61–2.43)	0.567		
Marital status					
Married	93 (81.6)	Ref			
Single/widowed/divorced	21 (18.4)	1.19 (0.60–2.38)	0.619		
Living arrangement					
With spouse	92 (80.7)	Ref			
Without spouse	22 (19.3)	1.11 (0.57–2.17)	0.756		
Family history of malignancy					
No	74 (64.9)	Ref		Ref	
Yes	40 (35.1)	1.43 (0.81–2.51)	0.214	1.60 (0.89–2.88)	0.113
Family history of colorectal cancer					
No	99 (86.8)	Ref			
Yes	15 (13.2)	1.17 (0.53–2.58)	0.705		
CAM consumption/service before presentation					
No	99 (86.8)	Ref			
Yes	15 (13.2)	1.56 (0.67–3.64)	0.303		
Distance to the first health facility visited (km)					
≤3.8	62 (54.4)	Ref			
>3.8	52 (45.6)	0.88 (0.52–1.49)	0.644		
Insurance status					
National/private health insurance	58 (50.9)	Ref			
Out-of-pocket	56 (49.1)	1.09 (0.65–1.84)	0.738		
Number of red flag symptom(s) at presentation					
≥1 symptom(s)	43 (37.7)	Ref			
None	71 (62.3)	0.74 (0.43–1.28)	0.285		

Abbreviations: OR=odds ratio; CI=confidence interval; Ref=reference; CAM=complementary and alternative medicines

**Table 3.** Univariate and multivariate analyses of factors associated with diagnosis delay of ≥4 months in patients with CRC (*n* = 227).

Variables	Diagnosis delay >4 months (%)	Unadjusted OR (95% CI)	*p*-value	Adjusted OR (95% CI)	*p*-value
Age at diagnosis					
<60 years	81 (72.3)	Ref		Ref	
≥60 years	31 (27.7)	0.46 (0.27–0.81)	0.006	0.52 (0.28–0.95)	0.035*
Sex					
Female	63 (56.3)	Ref			
Male	49 (43.7)	0.98 (0.58–1.65)	0.928		
Monthly income (IDR)					
≤2,000,000	63 (56.2)	Ref			
>2,000,000	49 (43.8)	0.82 (0.48–1.38)	0.455		
Formal education attainment					
Higher degree	29 (25.9)	Ref			
Junior–senior high school	44 (39.3)	0.92 (0.48–1.76)	0.806		
Primary school or none	39 (34.8)	1.26 (0.64–2.51)	0.505		
Marital status					
Married	93 (83.0)	Ref			
Single/widowed/divorced	19 (17.0)	0.97 (0.49–1.93)	0.932		
Living arrangement					
With spouse	92 (82.1)	Ref			
Without spouse	20 (17.9)	0.92 (0.47–1.80)	0.805		
Family history of malignancy					
No	73 (65.2)	Ref			
Yes	39 (34.8)	1.39 (0.79–2.43)	0.256		
Family history of CRC					
No	94 (83.9)	Ref		Ref	
Yes	18 (16.1)	2.01 (0.88–4.57)	0.096	1.74 (0.70–4.31)	0.231
Distance to the first health facility visited (km)					
≤3.8	58 (51.8)	Ref			
>3.8	54 (48.2)	1.09 (0.65–1.83)	0.748		
Number of red flag symptom(s) at presentation					
≥1 symptom(s)	26 (23.2)	Ref		Ref	
None	86 (76.8)	2.73 (1.54–4.84)	0.001	2.73 (1.47–5.10)	0.002*
CAM consumption/service before diagnosis					
No	51 (45.5)	Ref		Ref	
Yes	61 (54.5)	2.33 (1.36–3.98)	0.002	2.01 (1.12–3.61)	0.019*
Number of health facility visits before diagnosis					
≤3	35 (31.2)	Ref		Ref	
>3	77 (68.8)	1.27 (0.72–1.82)	<0.001	3.51 (1.95–6.29)	<0.001*
Insurance status following presentation					
Non-incentivized/private	60 (53.6)	Ref			
Government-incentivized	52 (46.4)	1.13 (0.67–1.90)	0.655		

* *p* < 0.05. Abbreviations: OR = odds ratio; CI = confidence interval; Ref = reference; CAM = complementary and alternative medicines

**Table 4.** The association between the delays in the presentation and diagnosis and metastatic disease at diagnosis.

Delay parameter	Metastatic disease *n* (%)	OR (95% CI)	*p*-value
Presentation delay ^†^			
<1 month	54 (54.5)	Ref	
≥1 months	45 (45.5)	0.69 (0.41–1.18)	0.182
Diagnosis delay ^‡^			
<4 month(s)	40 (40.4)	Ref	
≥4 months	59 (59.6)	2.04 (1.17–3.53)	0.011*

†: Adjusted for age, sex, income and formal education attainment

‡: Adjusted for age, sex, income, formal education attainment and presentation delay

* *p*<0.05

Abbreviations: OR = odds ratio; CI = confidence interval

**Table 5.** Reasons for delay in the presentation of patients with CRC (*n* = 70).

Reasons	Frequency (%)
I thought it was not serious/cancer/could heal itself	34 (48.6)
I was afraid of seeing physician or going to healthcare facility	12 (17.1)
The symptoms did not caused me pain	10 (14.3)
I was concerned about the cost and not having insurance	9 (12.9)
I was afraid of possible diagnosis	7 (10.0)
I was afraid of undergoing surgery	6 (8.6)
I attempted to self-medicate by purchasing medicine at the pharmacy	5 (7.1)
I was too busy	2 (2.9)
I perceived the system to be intricate	2 (2.9)
I lacked information about this disease	2 (2.9)
I was afraid to going out due to COVID-19 pandemic	1 (1.4)

Abbreviations: COVID-19 = Coronavirus Disease 2019

## Supplementary information

**Supplementary Table 1.** Comparison of the duration (in days) of the presentation delay based on the subjects’ baseline characteristics.

Variables	Presentation duration median in days (Min–Max)	*Z* score/*X*^2 *^	*p*-value
Age at diagnosis			
<60 years	30.5 (0–2,092)	0.240	0.810
≥60 years	31 (0–899)		
Sex			
Female	23 (0–2,092)	–2.070	**0.038***
Male	46 (0–1,855)		
Monthly income (IDR)			
≤2,000,000	31 (0–2,092)	1.305	0.192
>2,000,000	25 (0–1,119)		
Formal education attainment			
Higher degree	14 (0–897)	4.224	0.121
Junior to senior high school	31 (0–2,092)		
Primary school or none	30.5 (0–899)		
Marital status			
Married	30 (0–2,092)	–0.659	0.510
Single/widowed/divorced	31 (0–899)		
Living arrangement			
With spouse	30 (0–1,119)	–0.626	0.531
Without spouse (with other or alone)	30 (0–2,092)		
Family history of malignancy			
No	30 (0–2,092)	–0.745	0.456
Yes	31 (0–1,119)		
Family history of CRC			
No	30 (0–2,092)	0.054	0.957
Yes	31 (0–897)		
CAM consumption/service before presentation			
No	30 (0–2,092)	–0.931	0.352
Yes	46 (0–838)		
Distance to the first health facility visited (km)			
≤3.8	31 (0–2,092)	0.272	0.786
>3.8	30 (0–1,855)		
Insurance status at first presentation			
National/private health insurance	30 (0–2,092)	–0.373	0.709
Out-of-pocket	31 (0–1,855)		
Number of red flag symptom(s) at presentation			
≥1 symptom(s)	45.5 (0–2,092)	1.379	0.168
None	30 (0–1,855)		
Stage at diagnosis			
I–III	36 (0–2,092)	1.185	0.236
IV	25 (0–899)		

* *Z*-score for Wilcoxon sum-rank test and chi-square for Kruskal Wallis test

Abbreviations: Min = minimum; Max = maximum; *X*^2^ = chi-square; CAM = complementary and alternative medicines

**Supplementary Table 2.** Comparison of the duration (in days) of the diagnosis delay based on the subjects’ baseline characteristics.

Variables	Diagnosis duration median in days (Min-Max)	*Z* score/*X*^2^	*p*-value
Age at diagnosis			
<60 years	151.5 (4–5,088)	2.837	**0.005***
≥60 years	90 (3–1,559)		
Sex			
Female	118 (3–1,427)	–0.337	0.736
Male	117 (6–5,088)		
Monthly income (IDR)			
≤2,000,000	127.5 (6–1,427)	0.969	0.333
>2,000,000	112 (3–5,088)		
Formal education attainment			
Higher degree	116.5 (12–1,559)	2.372	0.305
Junior to senior high school	107 (3–5,088)		
Primary school or none	151.5 (6–1,427)		
Marital status			
Married	118.5 (3–5,088)	0.639	0.523
Single/widowed/divorced	115 (8–1,067)		
Living arrangement			
With spouse	119 (3–5,088)	0.683	0.494
Without spouse (with other or alone)	116.5 (4–1,067)		
Family history of malignancy			
No	108.5 (6–1,276)	–1.100	0.272
Yes	141 (3–5,088)		
Family history of CRC			
No	110 (3–1,559)	–2.717	**0.007***
Yes	272.5 (21–5,088)		
Distance to the first health facility visited (km)			
≤3.8	115 (3–5,088)	–0.235	0.814
>3.8	125 (4–1,599)		
Number of red flag symptom(s) at presentation			
≥1 symptom(s)	89 (4–1,095)	–2.774	**0.005***
None	148 (3–5,088)		
CAM consumption/service before diagnosis			
No	90 (3–1,087)	–4.377	**<0.001***
Yes	181 (6–5,088)		
Number of health facility visits before diagnosis			
≤3	71 (3–1,559)	–5.112	**<0.001***
>3	182 (6–5,088)		
Insurance status following presentation			
Non-incentivized or private	115 (4–5,088)	–0.269	0.788
Government-incentivized	125 (3–1,276)		
Stage at diagnosis			
I–III	93.5 (4–5,088)	–2.824	**0.005***
IV	154 (3–1,559)		

* *Z*-score for Wilcoxon sum-rank test and chi-square for Kruskal Wallis test

Abbreviations: Min = minimum; Max = maximum; *X*^2^ = chi-square; CAM = complementary and alternative medicines

**Supplementary Table 3.** Reasons for the delayed presentation in all the respondents who waited for ≥1 month to visit a health care facility (*n* = 99).

Reasons	Frequency (%)
I thought it was not serious/cancer/could heal itself	52 (52.5)
The symptoms did not caused me pain	18 (18.2)
I was afraid of seeing physician or going to healthcare facility	14 (14.1)
I was afraid of undergoing surgery	10 (10.1)
I was concerned about the cost	9 (9.1)
I was afraid of possible diagnosis	8 (8.1)
I attempted to self-medicate by purchasing medicine at the pharmacy	6 (6.1)
I perceived the system to be intricate	6 (6.1)
I was too busy	6 (6.1)
The health facilities were distant	3 (3.0)
I was afraid to going out due to COVID-19 pandemic	2 (2.0)

Abbreviations: COVID-19 = Coronavirus Disease 2019
